# Towards an accurate and robust analysis pipeline for somatic mutation calling

**DOI:** 10.3389/fgene.2022.979928

**Published:** 2022-11-15

**Authors:** Jingjie Jin, Zixi Chen, Jinchao Liu, Hongli Du, Gong Zhang

**Affiliations:** ^1^ Key Laboratory of Functional Protein Research, Guangdong Higher Education Institutes, Jinan University, Guangzhou, China; ^2^ MOE Key Laboratory of Tumor Molecular Biology, Institute of Life and Health Engineering, Jinan University, Guangzhou, China; ^3^ School of Biology and Biological Engineering, South China University of Technology, Guangzhou, China; ^4^ Chi-Biotech Co. Ltd., Shenzhen, China

**Keywords:** somatic mutation analysis, algorithm, accuracy, performance, next-generation sequencing—NGS

## Abstract

Accurate and robust somatic mutation detection is essential for cancer treatment, diagnostics and research. Various analysis pipelines give different results and thus should be systematically evaluated. In this study, we benchmarked 5 commonly-used somatic mutation calling pipelines (VarScan, VarDictJava, Mutect2, Strelka2 and FANSe) for their precision, recall and speed, using standard benchmarking datasets based on a series of real-world whole-exome sequencing datasets. All the 5 pipelines showed very high precision in all cases, and high recall rate in mutation rates higher than 10%. However, for the low frequency mutations, these pipelines showed large difference. FANSe showed the highest accuracy (especially the sensitivity) in all cases, and VarScan and VarDictJava outperformed Mutect2 and Strelka2 in low frequency mutations at all sequencing depths. The flaws in filter was the major cause of the low sensitivity of the four pipelines other than FANSe. Concerning the speed, FANSe pipeline was 8.8∼19x faster than the other pipelines. Our benchmarking results demonstrated performance of the somatic calling pipelines and provided a reference for a proper choice of such pipelines in cancer applications.

## Introduction

Somatic mutation is a key to provide insights and treatment of cancer. Most targeted cancer therapies are targeting specific somatic mutations, such as EGFR, BRAF, VEGF and BRCA mutations in various cancers (Gerlinger et al., 2012; Hirsch et al., 2017). Somatic mutation of circulating tumor DNA (ctDNA) may also serve as an indicator of cancer and its progression (Rolfo et al., 2021). Recently, the somatic mutation-derived cancer neoantigens become potential targets for immunotherapy (Ma et al., 2022). Therefore, accurate detection using next-generation sequencing (NGS) at genome-wide scale is crucial in cancer clinical applications. Although many analysis pipelines have been developed, the accuracy of somatic mutation detection is still a great problem. For example, a study showed zero sensitivity in finding pathogenic mutations using whole exome sequencing of 57 patients (Park et al., 2015). The mutation of 40 ctDNA samples, sequenced by two individual companies, showed only 12% congruence (Torga and Pienta, 2018). Such a low reproducibility illustrated the well-known “alarming reproducibility crisis” (Nekrutenko and Taylor, 2012).

When the specimen collection and experimental processes are standardized and quality-controlled, getting high quality sequencing raw data is expected. However, different computational pipelines can produce significantly different results. Many studies exhibited low concordance of variant-calling pipelines (Park et al., 2015; O'Rawe et al., 2013). The analysis pipeline normally contains two steps: mapping and somatic mutation calling. There are many algorithms available for each step. Users often randomly choose tools for analysis, creating a chaos in the field. Most benchmarking efforts used simulated datasets to test various pipelines (Wang et al., 2013; Ewing et al., 2015; Kroigard et al., 2016), but their conclusions hardly match, probably because the features of the simulated datasets vary. This also indicated that their performance in the simulated datasets may not necessarily reflect the performance in real-world applications. A few approaches used datasets of clinical samples (Alioto et al., 2015). However, it is almost impossible to yield true set for somatic mutations at genome-wide scale. In another aspect, with the rapid decreasing NGS experimental cost, the computational cost becomes a major part. The more and more sophisticated variant-calling algorithms often implements multiple filter steps, leading to prolonged running time. However, the clinical applications usually need the analysis as fast as possible. Therefore, it is also important to investigate how to accelerate the analysis when ensuring the accuracy.

Various somatic mutation detection pipelines have been developed, such as VarScan (Koboldt et al., 2012), VarDictJava (Lai et al., 2016), Mutect2 (in GATK (McKenna et al., 2010)), Strelka2 (Kim et al., 2018) and FANSe (Zhang et al., 2021). All these pipelines have been successfully used in clinical cases to detect specific cancer mutation, especially driver mutations (Welch et al., 2012; Desai et al., 2018; Lin et al., 2020; Mathioudaki et al., 2020; Farswan et al., 2021). Although single mutations can be experimentally validated by other methods, the accuracy of genome-wide mutation detections needs to be systematically evaluated using standard benchmarking datasets with known mutation results (“true set”). Chen et al. generated somatic mutation benchmarking datasets using real-world data (Chen et al., 2020). They mixed the whole-exome sequencing (WES) datasets of two normal human cell lines together to generate test datasets, and provided somatic mutation true sets using the germline variations, which is relatively easy to get. Therefore, such benchmarking datasets are representative. In this study, we evaluated the abovementioned 5 somatic mutation detection pipelines for their accuracy (precision and recall) and speed, at various mutation rates and sequencing depths.

## Materials and methods

### Datasets and true sets

Standard somatic mutation benchmarking files were taken from Chen et al. (Chen et al., 2020). Sequencing depth: 100 ×, 200 ×, 300 ×, 500 ×, 800 ×. Mutation rates: 1%, 5%, 10%, 20%, 30%, and 40%. For each configuration (depth and mutation rate), three independent files were used. The true sets for each dataset were also taken from Chen et al. (Chen et al., 2020)

### Hardware

For the speed test, all analyses were run in a workstation with AMD Threadripper 1950X CPU (16 cores, 32 threads) and 128GB RAM.

### Calling somatic mutations

BWA-MEM (Li and Durbin, 2009) and FANSe3 (Zhang et al., 2021) were used to map raw reads to the human reference genome hg19. The BWA-MEM were set to default parameters, and FANSe3 was set to “5% error tolerance, indel detection on, unique mapped reads only”.

In the SNV (single nucleotide variation) detection stage, the major parameters of the pipelines were set as follows:

**VarDictJava(v1.8.3)**: “-f 0.01” was both set when runing VarDict program and var2vcf_paired.pl script including in VarDictJava.
**VarScan(v2.4.2)**: “--min-coverage 1 --min-reads2 1 --min-var-freq 0.001” was set when running pileup2snp subprogram to call SNV, then “--min-coverage-tumor 3 --min-coverage 3 --min-coverage-normal 3 --min-var-freq 0.001 --somatic-p-value 1” was set when running somatic subprogram to call somatic variants.
**Mutect2(v4.1.0.0, v4.1.5.0, v4.2.0.0, v4.2.5.0)**: parameter “disable-read-filter” was set to “MateOnSameContigOrNoMappedMateReadFilter”.
**Strelka2(2.9.10)**: parameter “--exome” was set.
**FANSe3 (v3.12, commercial version)**: minimum coverage was set to 3.

**FIGURE 1:**
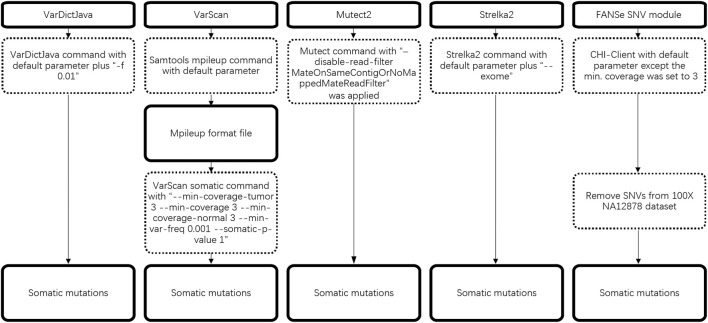
Workflow of 5 somatic mutation pipelines.

The detailed workflow of somatic mutation is illustrated in Figure 1.

Somatic mutations exported by all 5 pipelines were then compared to the true mutation set. The precision, recall and F-score were defined as Chen et al. (Chen et al., 2020) In brief, Precision rate, recall rate and F-score were defined as TP/(TP + FP), TP/(TP + FN) and 2*recall*precision/(recall + precision), respectively, where TP = true positives, FP = false positives, FN = false negatives.

## Results

### Running speed of the pipelines

In clinical practice, hundreds of cancer samples can be sequenced in one batch due to the capability of modern next-generation sequencers, creating massive computational workloads. Therefore, running speed can be a limiting factor of the entire pipeline. In general, the analysis time comprises three major parts: network transfer/decompression time, mapping time and SNV calling time.

**FIGURE 2:**
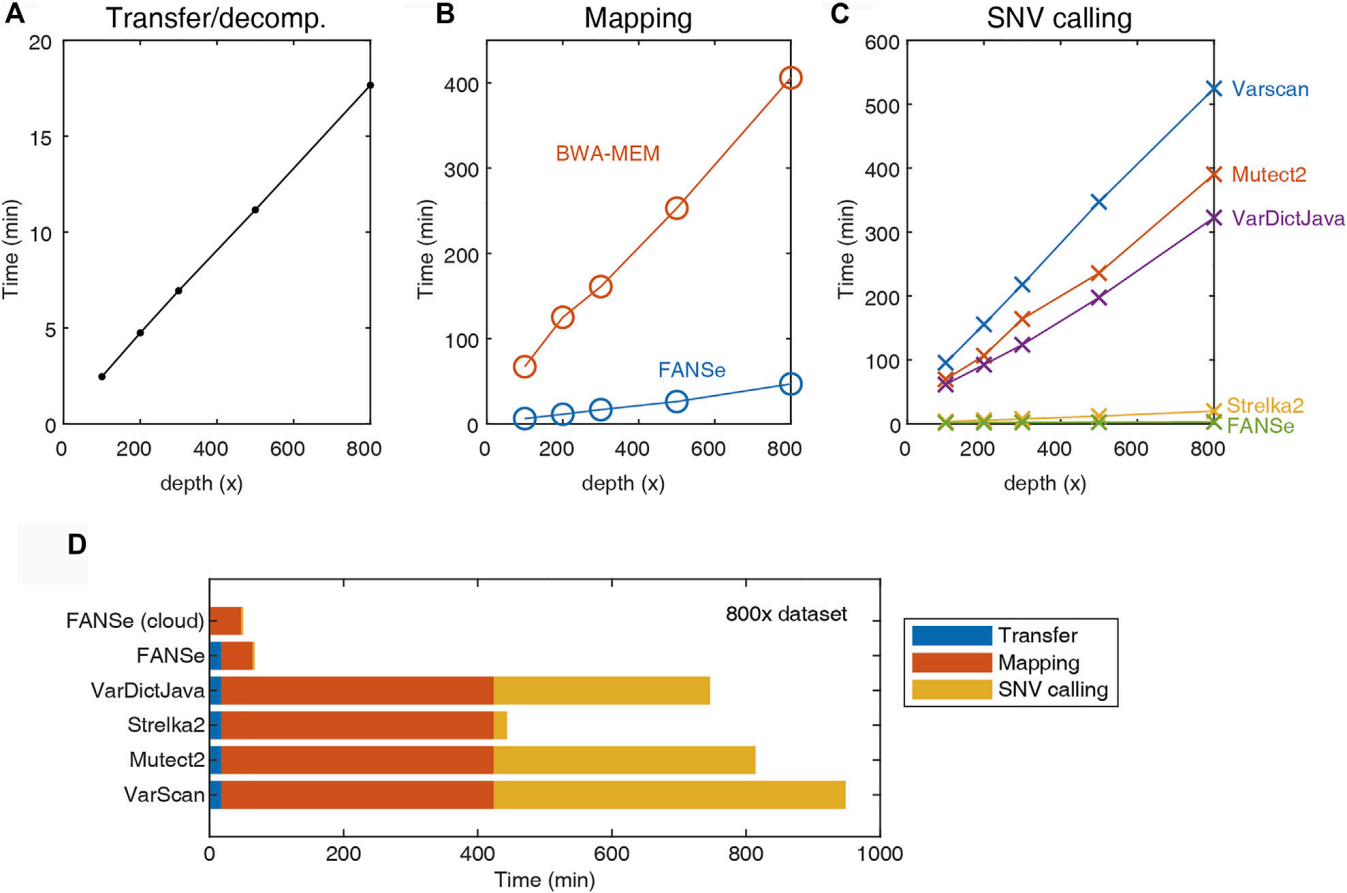
The speed of the 5 pipelines tested on an AMD Threadripper 1950X computer with 128G RAM. **(A)** Transfer (gigabit LAN) and decompression time of the datasets. **(B)** The mapping time of FANSe3 and BWA-MEM algorithms. **(C)** The SNV calling time. **(D)** The sum of running time of 5 pipelines.

Due to the large file size, transferring and decompressing the FASTQ files from storage server to the analysis server requires considerable time (Figure 2A). The mapping was performed using the BWA-MEM and FANSe3 algorithms, respectively. The speed of FANSe3 was almost 8 times faster than BWA-MEM (Figure 2B). In the SNV-calling stage, Mutect2, Strelka2, Varscan and VarDictJava accepted the .bam files from BWA-MEM, while FANSe needs its specific SNV module to call SNVs due to its special output format (Figure 2C). There are enormous differences between the speed of these pipelines. Strelka2 used ∼20 min to finish the SNV calling for the 800 × WES dataset, which was 20 times faster than Varscan, Mutect2 and VarDictJava. However, FANSe SNV module used only less than 3 min to finish the SNV calling, 6.7 times faster than the Strelka2 and 131 times faster than Mutect2. When summing all the running times together, the FANSe solution showed great advantage in speed (Figure 2D). When deployed in cloud-computing infrastructure, FANSe can be configured to do the mapping when the file transfer is going on. This would further save the time of transfer and thus decrease the total time of processing (Figure 2D).

### Recall and precision of the pipelines

**FIGURE 3:**
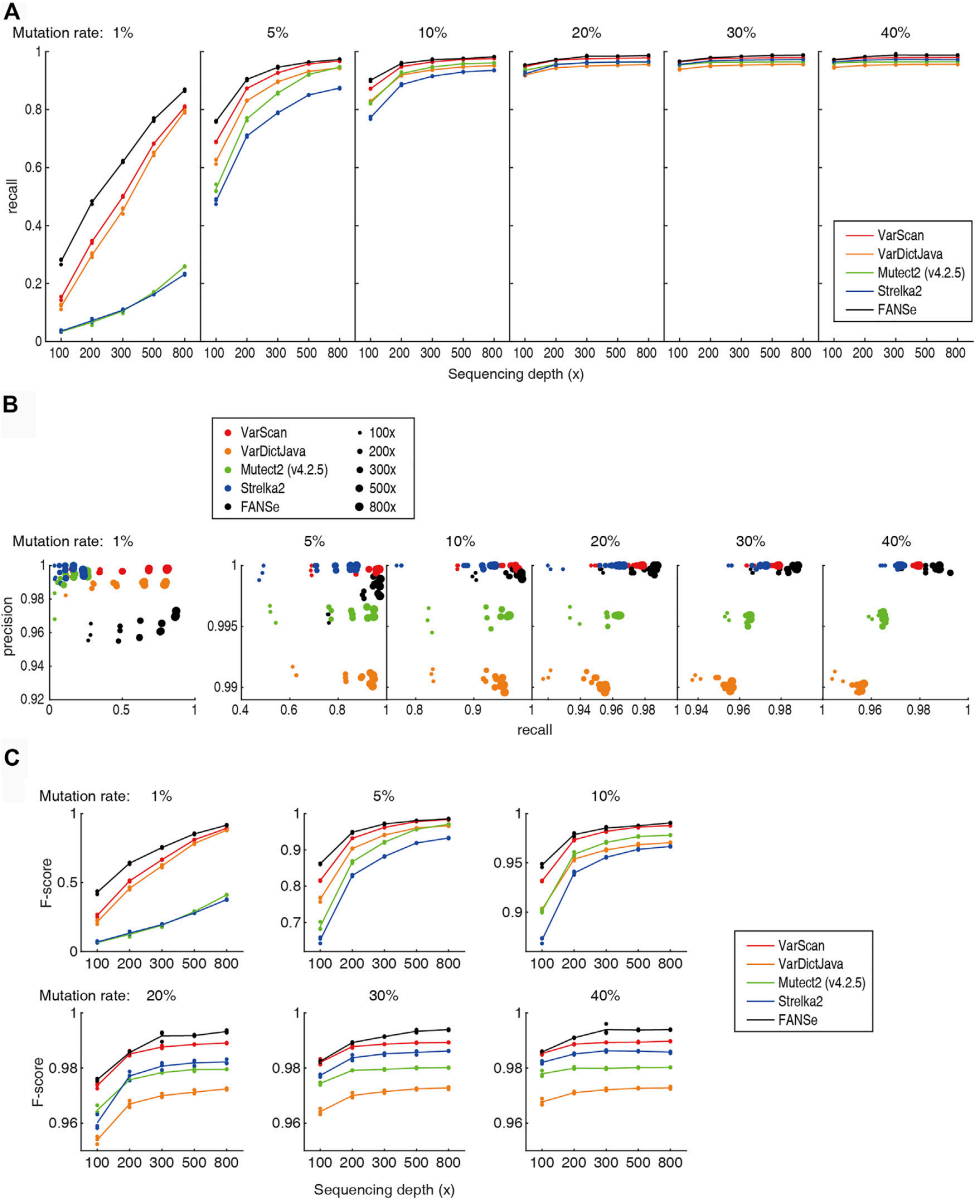
Accuracy of the 5 pipelines. All detailed numbers are listed in Supplementary Table S1. **(A)** The recall rate of the 5 pipelines at different sequencing depths and mutation rates. For each mutation rate and sequencing depth, the data points for the 3 datasets were all shown, and the lines illustrate their mean values. **(B)** The precision-recall diagram of the 5 pipelines. Color indicates the pipeline, and the dot size indicates the sequencing depths. **(C)** The F-scores of the 5 pipelines.

Previous study showed that the Mutect2 and Strelka2 pipelines could not effectively detect the low frequency somatic mutations even at 800x sequencing depth (Chen et al., 2020), which prohibited them from clinical practice. However, after excluding the PCR duplicates (e.g., using UMI), a specific nucleotide mutation should be detectable given high enough sequencing depths. Theoretically, at sequencing depth of 800 × and 0.5% sequencing error, 1% mutation rate should be significantly detected (*p* = 0.021, Fisher exact test). Therefore, we believe that there should be algorithms that can reliably detect most of the 1% somatic mutations at high sequencing depth. Indeed, the recall rate of FANSe, VarDictJava and VarScan was overwhelming against Mutect2 and Strelka2 (Figure 3A). For 800 × datasets, FANSe reached ∼87% recall rate, and VarDictJava and VarScan reached 81% and 79% in average, respectively. This performance was already informative. In contrast, Mutect2 and Strelka2 reached 26% and 23%, respectively, even lower than the recall rate of FANSe at 100 × sequencing depth (28%). This validated our hypothesis that proper algorithm can effectively detect low frequency somatic mutations.

At all sequencing depths and mutation rates, FANSe’s recall rate was higher than VarDictJava and VarScan. For example, for 1% and 5% mutation rates, FANSe at 200 × depth was comparable to VarDictJava and VarScan at 300 ×. This indicated that using FANSe lowers the need of sequencing throughput, i.e., reduces the sequencing cost.

The precision of all pipelines were quite high (Figure 3B). At 1% mutation rate, the precision of all pipelines were greater than 95%, and in higher mutation rates, the precision were all greater than 99% except VarDictJava. At 1% mutation rate, FANSe showed the best recall rate but slightly lower precision than VarDictJava and VarScan. However, the precision of FANSe rapidly increased when the mutation rate was higher. When mutation rate was higher than 5%, Mutect2 and VarDictJava showed remarkable disadvantage in precision and recall. When assessed by the criteria F-score (Figure 3C), FANSe was the best in all cases, and the VarScan ranked the second. VarDictJava performed quite well at 1% and 5% mutation rates, but was surpassed by Mutect2 and Strelka2 at higher mutation rates.

### Version advances cause remarkable difference in results

**FIGURE 4:**
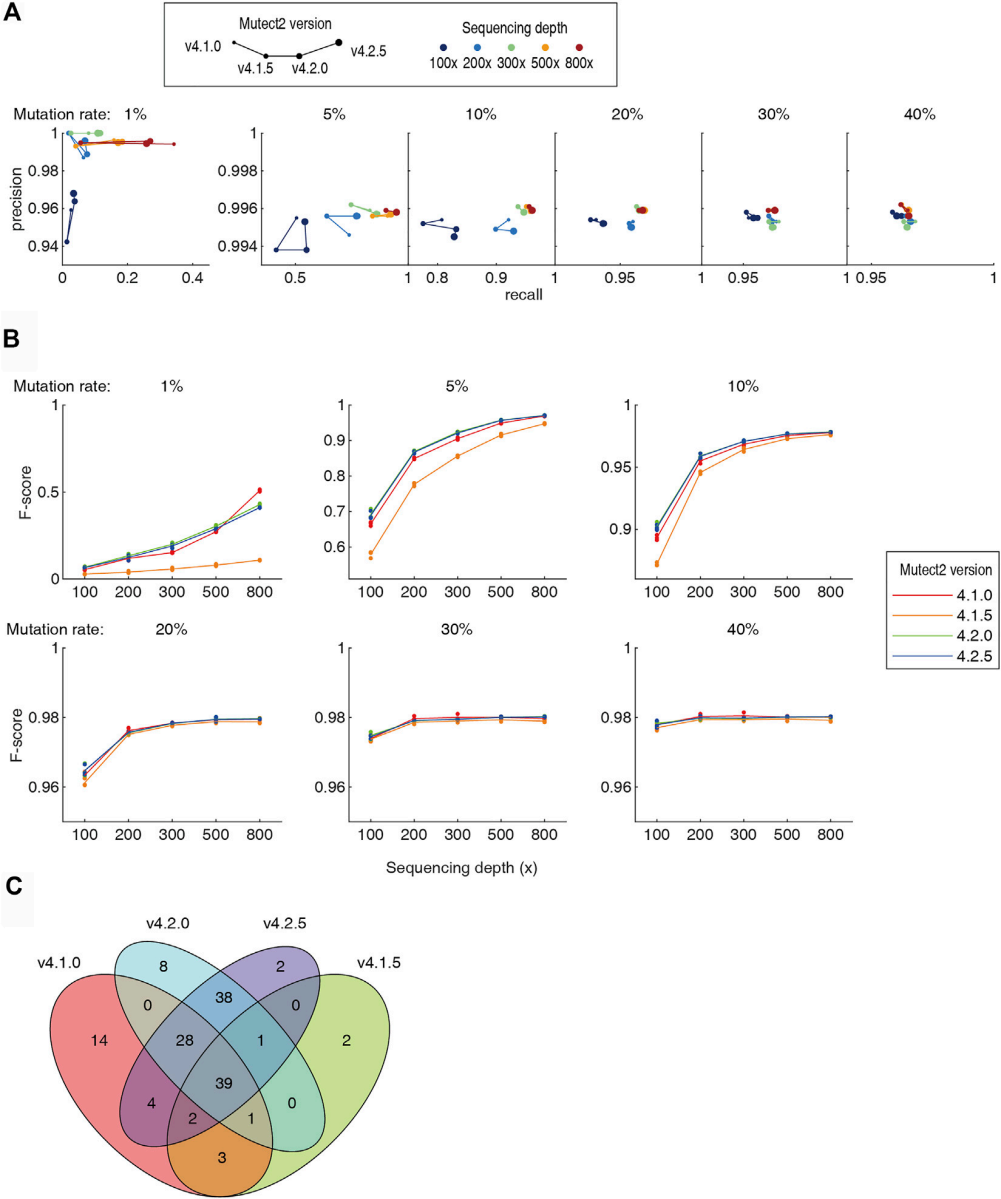
Comparison of four versions of the Mutect2. Detailed numbers were listed in Supplementary Table S1. **(A)** The precision-recall diagram of Mutect2 v4.1.0 to v4.2.5. The size of dots indicates the version, and the color indicates the sequencing depths. For each sequencing depth and each mutation rate, only one dataset was chosen to make the diagram for visibility. **(B)** The F-scores of the four versions of the Mutect2. **(C)** The Venn diagram of the somatic mutations detected by four versions of the Mutect2.

It has been noted since decade that the different version of the same software can generate totally different results when analyzing the same NGS dataset, contributing to the “reproducibility crisis”. In the 5 pipelines tested in this study, Mutect2 was constantly updated for newer versions. We tested the Mutect2 versions 4.1.0, 4.1.5, 4.2.0 and 4.2.5 on the benchmarking datasets. The v4.1.5 showed a remarkable drop in both recall rate and precision (Figure 4A). Newer version did not necessarily provide better recall and precision. For higher sequencing throughput and/or higher mutation rate (≥5%), the version-dependent variation was in general smaller. Assessed using the F-score, not a single version was the best in all cases (Figure 4B). For example, v4.1.0 had the best F-score at 30–40% mutation rates and 200–300 × depth, and had a large advantage against all later versions at 1% mutation rate and 800 × depth. The newest v4.2.5 was not the best at all mutation rates and 800 × depth. Moreover, the somatic mutations called by different versions differ largely. As an example, for a dataset of 1% mutation rate and 100x depth, v4.2.0 and v4.2.5 screened 115 and 114 somatic mutations, respectively. 93% of them overlap. However, the consistency of the v4.2.5 and v4.1.0 was only 64% (Figure 4C). Although algorithmic improvements may be implemented during the version iterations, such a high inconsistency set a warning that the users must be aware the discrepancy caused by the software versions.

### Filtering is a key of SNV detection

**FIGURE 5:**
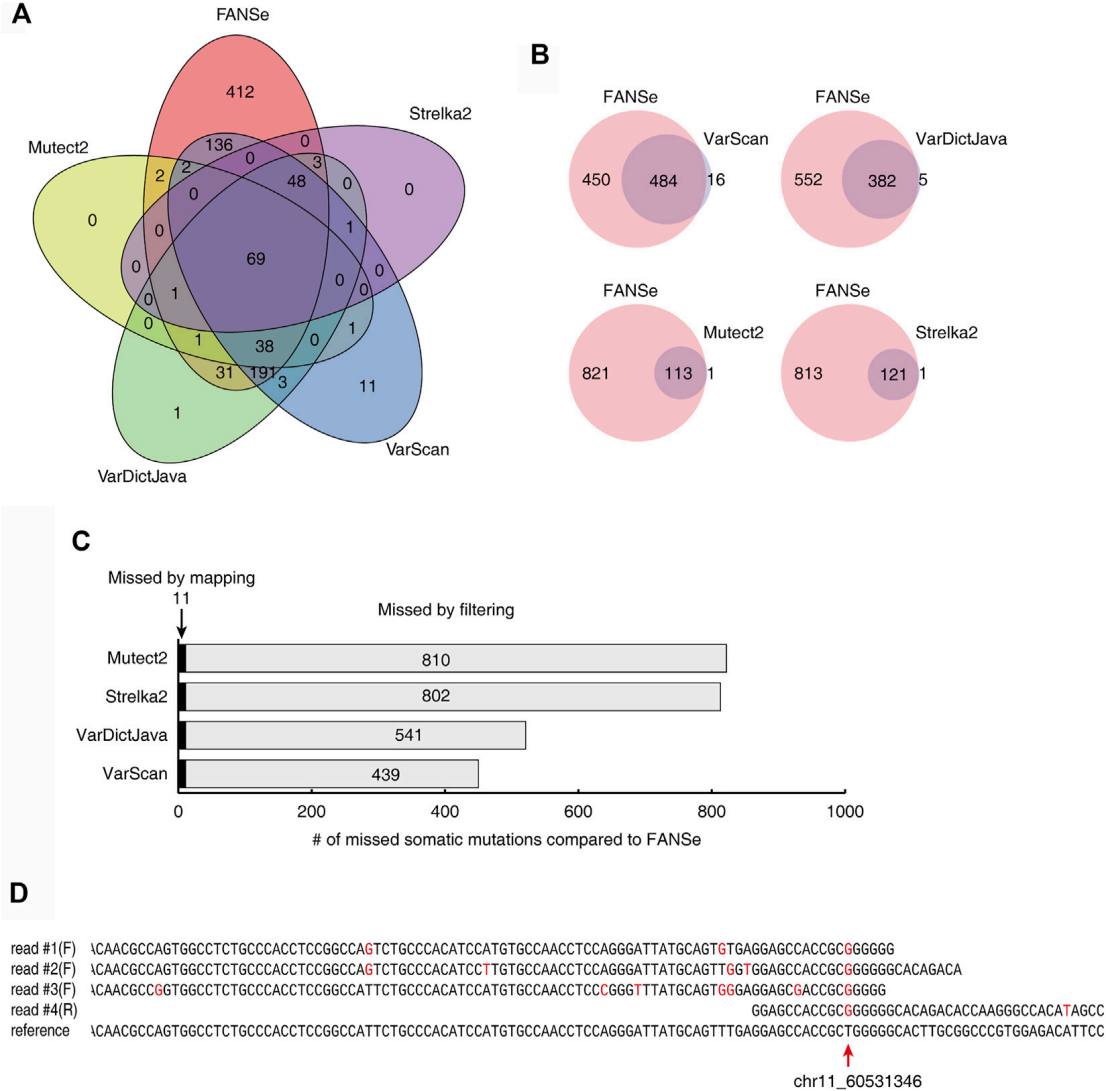
Comparison of the somatic mutations identified by 5 pipelines on the dataset #1 of 1% mutation rate, 100x sequencing depth. **(A)** The Venn diagram of the somatic mutations identified by the 5 pipelines. **(B)** The somatic mutations identified by FANSe pipeline and the other pipelines, respectively. **(C)** The reason of the missed mutations by the other 4 pipelines compared to FANSe. **(D)** An example of mutation site (chr11, position 60531346), where the mismatch-containing reads were not mapped by BWA-MEM. Four mismatch-containing reads were mapped by FANSe and piled on the reference sequence. The read #1∼#3 were mapped to the forward strand and the read #4 was mapped to the reverse strand (illustrated as reverse-complement). Red bases illustrated the mismatches against the reference sequences.

The somatic mutations detected by the 5 pipelines showed very little consistency. For a dataset of 1% mutation rate and 100 × depth, only 69 somatic mutations were identified by all 5 pipelines (Figure 5A). FANSe identified many more somatic mutations than the other pipelines, and the mutations identified by the other pipelines were almost covered by FANSe (Figure 5B). Considering the high precision of FANSe, the mutations that were solely identified by FANSe were almost all true. It is an interesting question why the other pipelines missed so many mutations. After comparison, we found that 11 mutations were missed by other pipelines because the BWA-MEM failed to map the mismatch-containing reads to the reference genome (Figure 5C, details listed in Supplementary Table S2). Most of such reads contained multiple mismatches against the reference sequences (an example was illustrated in Figure 5D), indicating that the BWA-MEM mapping algorithm is not robust enough to map such reads. The rest 439∼811 (>97.5%) missed mutations were detected by the SNV-calling algorithms but were missed because the algorithms filtered them out (Figure 5C). Obviously, these filters need to be improved. However, the details of these filters were not clear in the literatures, prohibiting us to raise suggestions to improve.

## Discussion

Taking the advantage of the standard benchmarking WES datasets, we can assess the performance of any somatic mutation detection pipelines. Chen et al. compared the Mutect2 and Strelka2 pipelines. They concluded that the sensitivity at low mutation frequency is inacceptable, and cannot be compensated by the elevation of sequencing depth. However, we found that many other pipelines like VarDictJava, VarScan and FANSe, showed several times higher sensitivity than Mutect2 and Strelka2. This indicated that there are many more sensitive algorithms available; however, most researchers, especially those who are not professional in bioinformatics, did not choose a proper analysis pipeline based on real-world data benchmarking. This algorithm blind pick may generate misleading results that confuse further investigations, and causes inconsistent conclusions, intensifying the “alarming reproducibility crisis”.

The good news from our study is the very high precision of all 5 tested pipelines: at 1% mutation frequency, the precision is above 95%, and at higher mutation frequency, the precision is nearly perfect. This means that the somatic mutations called by these pipelines were nearly true. However, the sensitivity is a major challenge, i.e., most pipelines missed a considerable fraction of somatic mutations. Considering only the computational analysis, flaws in two steps contribute to the loss of sensitivity: 1) The miss of the mapping algorithms. The insufficient robustness and error tolerance of the mapping algorithms failed to map some mismatch-containing reads to the reference genomes, especially when these reads also contains some germline mutations. In this study, we found that 1∼2.5% of the somatic mutations were missed by this reason, which is relatively minor. 2) The improper filter. Each SNV-calling algorithm has its distinguished statistical model, but is rarely publicized in detail. Therefore, it is hard for the users to suggest improvements of the model. In addition, the filter criteria are often changed during the version upgrade, leading to considerable inconsistency over the versions. We demonstrated such inconsistency in Mutect2. The researchers need to be aware of this. Some algorithms like Mutect2 contain many filter steps, creating complexity that might reduce robustness when compared to simpler models like VarScan.

FANSe pipeline showed distinct advantage in both accuracy and speed. It has the best F-score in all cases, especially at low mutation frequency and low sequencing depth. At 1% mutation rate, its sensitivity reached 87% for 800 × sequencing depth, which is quite useful in clinical practice. At 5% mutation rate, its sensitivity reached 95% for 300 × depth; increasing the sequencing depth to 800 × only slightly increased the sensitivity to 97%. At 10% mutation rate, 200 × depth almost reaches the stationary level of sensitivity, and more throughput hardly benefits. This means that the sensitive and accurate somatic mutation detection can be achieved at relatively low sequencing cost. Also, its speed is 8.8 times faster than the Strelka2, and 19 times faster than VarScan, which showed a great advantage in the computational efficiency–and cost. It may be the optimal solution for somatic mutation detection in the precision medicine era.

## Data Availability

The original contributions presented in the study are included in the article/Supplementary Material, further inquiries can be directed to the corresponding authors.
